# Practical Notes and Formulæ

**Published:** 1881-03-20

**Authors:** 


### PRACTICAL NOTES AND FORMULÆ.

**For Vomiting in Pregnancy**—Dr. Goodell recommends:

℞ Cerii oxalatis,........................................ } aa gr. j.

Ipacacuanhæ,........................................ }

Creosoti,........................................ gtt. ij.

M. Sig. To be taken every hour.—*Med. Gaz.*

**Jamaica Dogwood in Treatment of Pertussis.**—Dr. W. R. Alexander, of West Virginia, in Therapeutic Gazette, is quite enthusiastic in his recommendation of this new drug. He says the effect in whooping-cough was quite satisfactory, and in his hands it proved a specific in a number of cases. He now orders it in this disease with as much confidence in its results as he does quinine in malarial affections. Sustaining the patient's strength by stimulants and nourishment, he gives it to children at all ages and in any stage of the fever. The initial catarrh and the catarrhal stages are decidedly benefitted, and the spasmodic attack in many cases wholly aborted.

He recommends the following prescription:

℞ Fl. ext. Jamaica dogwood,........................................ ʒjss.

Syr. tolu........................................ } aa ℥ ij.

Syr. acaciæ,........................................ }

M. Sig. Teaspoonful every two, three or four hours, according to the violence of the spasm or cough. A few drops of the fluid extract in a teaspoonful of water may also be given with good results.—*Louisville Medical News.*

**Dysmenorrhœa**—Dr. Pearson, in Medical and Surgical Reporter, says: I have, for some time past, treated cases of dysmenorrhœa as follows, with very satisfactory results—

℞ Iodidi potassii,........................................ } aa ʒj.

Bromide potassii,........................................ }

Syr. simp.,........................................ ℥ iij.

A teaspoonful to be taken three times daily, commencing ten days previous to menstrual period.

When pain commences—

℞ Tinct. opii. camph........................................ ℥ ij.

Morph. sulph........................................ gr. ij.

Ol. piper........................................ gtt. ij.

A teaspoonful every three or four hours while pain continues.

The above has proved very satisfactory to me and may be worth something to others, as I have seldom had to repeat the course more than once to effect a permanent cure.

**Removal of Nævus.**—Dr. Sigler states, in the Pharm. Centralb., that nævus may be removed by means of croton oil, in the following manner: Push a number of needles through a cork, so that the points project 3 to 4 millimetres. Dip the points in croton oil, then insert them in the mole and withdraw. This is a sort of Baunscheidtismus. A scab will form upon the mole; and after it has dried up and dropped off the operation is twice more repeated.

**Chemistry of Common Life.**—Dr. J. Hendree, of Alabama, writes: Ought not teachers in common and other schools to have some knowledge of the chemistry of every-day life? I read, recently, that a female teacher in a Pennsylvania school put wood ashes in the mouth of a child six years old to punish him, she said, for telling a lie. Is a person competent to teach who does not know the common fact, familiar to an Alabama negro, that ashes and water will form caustic ley? Of course, the child's mouth, lips and throat were perfectly excoriated. Comment is unnecessary.

I believe the education of our children in the physical sciences, especially the chemistry of common life, should be commenced at an early age, with plain, cheap apparatus and simple, attractive experiments. Knowledge thus inculcated is not forgotten.

**Gastritis.**—Dr. R. S. Forehand, of Georgia, suggests the following treatment for gastritis: The first indication of treatment in this, as in all other affections, is to remove the exciting cause; first stop the vomiting and open the bowels by injections of warm water; after the bowels are open put a large blister over the stomach, also take sixty grains of chlorate of potash put in four ounces of water, give one teaspoonful every two hours. It will be advisable not to give any other medicines by the mouth, except calomel. It is well borne by the stomach, as long as acute inflammation exists. You can give small doses of Dover's powder every two or three hours to keep your patient quiet. Also, if the skin is hot and dry, give ten or fifteen drops of spirits of nitre every three hours. If your patient would like to eat something, let it be light, such as milk punch every three or four hours.

**Acute Catarrh.**.—

| | | |
|---|---|---|
| ℞ | Tinct. iodinii | ℥ ss. |
| | Acid carbol | ʒ j. |

M. Sig. Place a small, wide-mouthed bottle, containing a moistened sponge, in a vessel of hot water; drop five to ten drops of the solution on the sponge and as the iodine vapor ascends with the vapor of the water inhale it.—*Med. Gaz.*

**A Stimulating Expectorant**—

| | | |
|---|---|---|
| ℞ | Am. carbonat | gr. v. |
| | Tinct. nux vom | m. x. |
| | Tinct. scillæ | ʒ ss. |
| | Inf. serpentar | ℥ ij. |

M. Sig. Three times a day.—*Fothergill.*

In those cases in which chronic bronchitis is associated with emphysema, or in the second stage of acute bronchitis, where the heart is severely taxed, this combination of remedies will strengthen the over-taxed heart and clear out the air passages.—*Ibid.*

**Special Affection Following Diphtheria.**—In the *Prag. Med. Wohmens et Lyon Medicale* a case of pneumonia and severe myalgia is reported following diphtheria, supposed to be dependent upon the migration or invasion of the diphtheritic parasites.

